# Assessment of various parameters to improve MALDI-TOF MS reference spectra libraries constructed for the routine identification of filamentous fungi

**DOI:** 10.1186/1471-2180-13-76

**Published:** 2013-04-08

**Authors:** Anne-Cécile Normand, Carole Cassagne, Stéphane Ranque, Coralie L’Ollivier, Patrick Fourquet, Sam Roesems, Marijke Hendrickx, Renaud Piarroux

**Affiliations:** 1 AP-HM, Parasitologie-Mycologie, CHU Timone, 13005, Marseille, France; 2 Aix-Marseille Université, UMR MD3, 13005, Marseille, France; 3 Service Proteomique, Centre d’Immunologie de Marseille Luminy, 13009, Marseille, France; 4 BCCM/IHEM: Scientific Institute of Public Health, Mycology and Aerobiology Section, Brussels, Belgium

**Keywords:** MALDI-TOF MS, Filamentous fungi, Molds, Reference spectra library

## Abstract

**Background:**

The poor reproducibility of matrix-assisted desorption/ionization time-of-flight (MALDI-TOF) spectra limits the effectiveness of the MALDI-TOF MS-based identification of filamentous fungi with highly heterogeneous phenotypes in routine clinical laboratories. This study aimed to enhance the MALDI-TOF MS-based identification of filamentous fungi by assessing several architectures of reference spectrum libraries.

**Results:**

We established reference spectrum libraries that included 30 filamentous fungus species with various architectures characterized by distinct combinations of the following: i) technical replicates, i.e., the number of analyzed deposits for each culture used to build a reference meta-spectrum (RMS); ii) biological replicates, i.e., the number of RMS derived from the distinct subculture of each strain; and iii) the number of distinct strains of a given species. We then compared the effectiveness of each library in the identification of 200 prospectively collected clinical isolates, including 38 species in 28 genera.

Identification effectiveness was improved by increasing the number of both RMS per strain (p<10^-4^) and strains for a given species (p<10^-4^) in a multivariate analysis.

**Conclusion:**

Addressing the heterogeneity of MALDI-TOF spectra derived from filamentous fungi by increasing the number of RMS obtained from distinct subcultures of strains included in the reference spectra library markedly improved the effectiveness of the MALDI-TOF MS-based identification of clinical filamentous fungi.

## Background

The identification of mold in the clinical laboratory is classically based on macroscopic and microscopic examination of the colonies grown on mycological culture media. It is a slow and complex process requiring highly skilled mycologists, and misidentifications may occur, even in experienced reference laboratories [1]. Additionally, some distinct species, which are identified via DNA sequence analysis, are morphologically indistinguishable [2-4]. Therefore, multilocus DNA sequence analysis represents the recommended approach to accurately identify these microorganisms. Nevertheless, the DNA sequence-based identification of filamentous fungi is primarily limited by the following: i) low DNA extraction yields because mold cells are difficult to lyse, ii) the presence of PCR inhibitors, iii) the presence of misidentified sequences in non-curated public DNA sequence databases, and iv) the cost and time required for sequencing. Currently, only some clinical laboratories routinely use a molecular approach for microorganism identification, which is primarily due to the cost and application constraints [5,6].

Recently, matrix-assisted desorption/ionization time-of-flight (MALDI-TOF) mass spectrometry (MS) has been applied to rapidly identify bacteria and yeasts in the clinical microbiology laboratory setting [7]. This technique is used to analyze microorganism content (primarily ribosomal proteins), thereby generating a spectrum that is considered the fingerprint of the microorganism [8]. Using this technique, the identification of an unknown organism is performed by comparing the corresponding spectrum to a reference library of spectra. When establishing a reference library for microbial identification purposes, many authors have used reference mass spectra, sometimes referred to as “metaspectra” or “superspectra”, which are generated by combining the results of a various number of individual spectra corresponding to technical replicates of a given sample. Previous studies have indicated that MS could be used to identify various filamentous fungi taxa of clinical interest, including *Fusarium* spp [9-11], dermatophytes [12,13], *Aspergillus* spp [14,15], and *Pseudallescheria*/*Scedosporium* spp [16]; those of industrial interest, including *Penicillium* spp [17,18], *Verticillium* spp [19], and *Trichoderma* spp [20]; and various filamentous fungal contaminants frequently isolated in the clinical laboratory [21,22].

**Figure 1:**
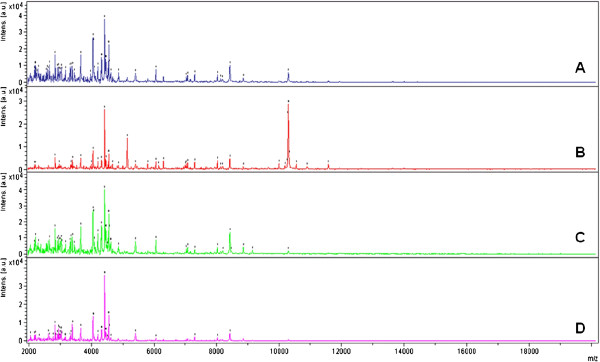
**Comparison of mass spectra obtained from four subcultures of a strain of *Aspergillus flavus.*** The *Aspergillus flavus* 1027804 strain was subcultured on four different agar plates. Spectra **A**, **B**, **C**, and **D** display the first spectrum acquired from the subcultures 1, 2, 3 and 4, respectively. Spectra A to D display many common peaks; however, a few varying peaks are also clearly visible and characteristic of one of the subcultures.

The heterogeneous morphological phenotypes of filamentous fungi affect the identification process. As shown in Figure 1, the same heterogeneity exists for MALDI-TOF mass spectra, between different strains of the same species as well as between subcultures of the same strain, which negatively impacts the reproducibility of the spectra. To troubleshoot this issue, we accounted for this heterogeneity during the establishment of the RMS library (MSL). We hypothesized that MS identification effectiveness could be enhanced by increasing both the number of reference meta spectra (RMS) of a given strain included in the reference library and the number of deposits used to generate each RMS. The primary objective of this study was to test the effectiveness of distinct reference spectra library architectures for the MALDI-TOF MS-based identification of filamentous fungi. More precisely, we assessed the influence on identification effectiveness of the following: i) the number of technical replicates, i.e., the number of analyzed deposits (spots) from one culture used to generate an RMS; ii) the number of biological replicates, i.e., the number of RMS derived from distinct subcultures for each strain; and iii) the number of distinct strains of one species used to construct the library.

## Results

### Phenotypic and genotypic identification of clinical isolates

The results of the classical and DNA sequence-based identification of 200 clinical isolates (Table 1) were applied to classify the isolates into two groups: isolates included and isolates excluded from the MSL. The MS results of both groups are summarized in Table 2. The isolates belonged to 28 different genera and 38 different species. Moreover, 174 isolates corresponded to 18 species, which were represented among those used to construct the eight libraries, whereas the 26 remaining isolates belonged to 20 species that were not represented in the libraries.

**Table 1:** Identification of the 200 clinical isolates included in the study

**Species**	**Number of Isolates**	**Corresponding RMS in the MSLs**
*Acremonium sp*.	1	no
*Alternaria alternata*	10	yes
*Aspergillus alliaceus*	2	no
*Aspergillus clavatus*	1	no
*Aspergillus flavus*	8	yes
*Aspergillus fumigatus*	82	yes
*Aspergillus melleus*	1	no
*Aspergillus nidulans*	1	yes
*Aspergillus niger*	12	yes
*Aspergillus oryzae*	1	no
*Aspergillus sydowii*	1	no
*Aspergillus terreus*	11	yes
*Beauveria bassiana*	1	yes
*Chaetomium globosum*	1	no
*Fomitopsis ostreiformis*	1	no
*Fusarium oxysporum*	2	yes
*Geotrichum candidum*	2	no
*Hamigera avellanea*	1	no
*Hypocrea jecorina*	1	no
*Microsporum audouinii*	10	yes
*Microsporum canis*	1	yes
*Onychocola canadensis*	1	no
*Penicillium aurantiogriseum*	1	yes
*Penicillium chrysogenum*	7	yes
*Penicillium citrinum*	1	no
*Penicillium funiculosum*	1	no
*Penicillium glabrum*	4	no
*Penicillium oxalicum*	1	no
*Penicillium pinophilum*	1	no
*Penicillium variotii*	1	yes
*Penicillum spinulosum*	2	no
*Rhizopus oryzae*	3	yes
*Scedosporium apiospermium*	8	yes
*Trichoderma atroviridae*	1	no
*Trichoderma viridae*	1	no
*Trichophyton mentagrophytes*	2	yes
*Trichophyton rubrum*	8	yes
*Trichophyton soudanense*	6	yes

RMS: reference mass spectrum in the mass spectra library (MSL) constructed from several raw spectra.

**Table 2:** **Details of the MS**-**based identification results of the 200 clinical isolates included in the study**

	**Mass spectra libraries**							
	**B0**	**B1**	**B2**	**B3**	**B4**	**B5**	**B6**	**B7**
**Isolates included in the MSLs (n**=**174)**								
Nb. of concordant identifications	481	449	495	521	494	475	586	611
Median value of concordant LS1 values	1.59	1.58	1.65	1.73	1.67	1.67	1.99	2.02
Nb. of concordant values with LS1>1.7	182	180	222	282	225	225	443	494
Percentage of concordant values with LS1>1.7	37.8	40.1	44.8	54.1	45.5	47.4	75.6	80.9
Range of concordant LS1 values	0.49 - 2.39	0.29 - 2.45	0.50 - 2.45	0.66 - 2.57	0.18 - 2.44	0.70 - 2.44	0.60 - 2.57	0.77 - 2.57
Nb. of non-concordant identifications	225	257	211	184	212	231	119	95
Median value of non-concordant LS1 values	0.99	1.07	1.1	1.23	1.15	1.07	1.26	1.28
Range of non-concordant LS1 values	0.29 - 1.44	0.14 - 1.55	0.27 - 1.58	0.43 - 1.58	0.25 - 1.85	0.14 - 1.52	0.65 - 1.69	0.69 - 1.69
**Isolates not included in the MSLs (n**=**26)**								
Nb. of concordant identifications	0	0	0	0	0	0	0	0
Median values of concordant LS1 values	-	-	-	-	-	-	-	-
Minimum and maximum values of the concordant LS1	-	-	-	-	-	-	-	-
Nb. of non-concordant identifications	104	104	104	104	104	104	104	104
Median values of non-concordant LS1 values	1.02	1.09	1.18	1.24	1.22	1.14	1.31	1.33
Range of non-concordant LS1 values	0.50 - 1.39	0.45 - 1.43	0.46 - 1.44	0.56 - 1.56	0.52 - 1.54	0.54 - 1.49	0.76 - 1.79	0.88 - 1.79

Concordant LS1: LS value for the first concordant identification with the library; non-concordant LS1: LS value for the first non-concordant identification with the library; Nb.: number.

### Reference MS library validation

**Figure 2:**
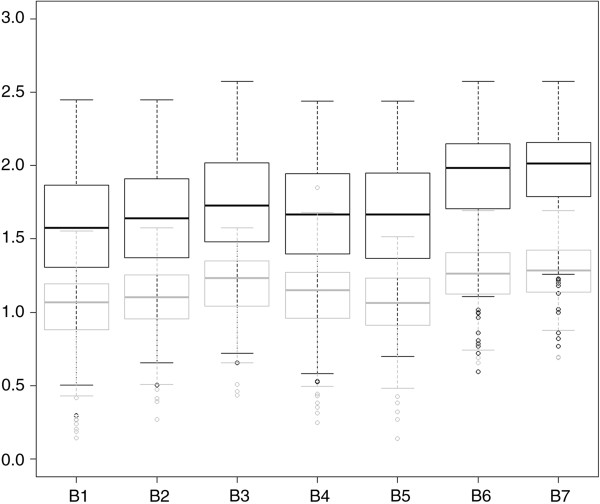
**Distribution of the LS1 values.** Box-and-whisker diagrams of the LS1 values associated with the concordant mass spectral identifications (black) and the non-concordant identifications (gray) obtained using the seven different mass spectrum libraries tested (B1 to B7). The lower and upper portions of the box represent the lower and upper quartiles, respectively. The dark band represents the median value. The ends of the whiskers represent the lowest datum included in the 1.5 inter-quartile range (IQR) of the lower quartile and the highest datum included in the 1.5 IQR of the upper quartile. Outlier values are represented by a circle; a.u.: arbitrary unit.

**Figure 3:**
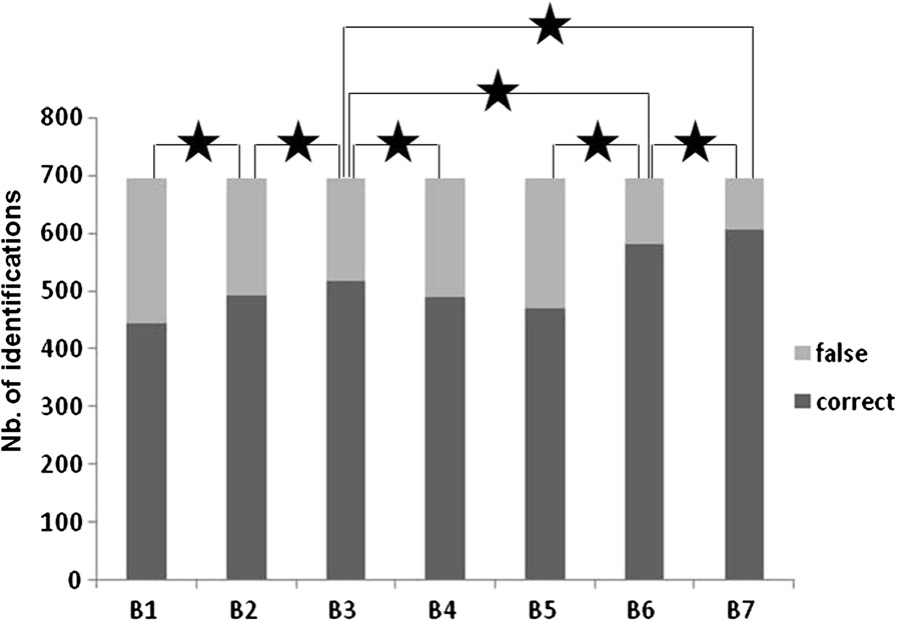
**Number of correct and false MALDI**-**TOF MS**-**based identifications obtained with the seven mass spectral libraries.** A bar graph showing the number of concordant and non-concordant MALDI-TOF MS-based identifications obtained with each of the seven different mass spectral libraries, B1 to B7, for the 174 isolates. The horizontal bar represents the significance of the McNemar’s test between the designated MSLs (★ p≤0.01; Nb.: number; MSLs, mass spectral libraries).

All 104 spectra derived from the 26 clinical isolates for which the species was not included in the seven MS libraries (4 raw spectra per clinical isolate) yielded low Log Scores (LS) ranging from 0.45 to 1.79 (only 1/104 spectra yielded LS>1.7: *Penicillium aurantiogriseum* identified instead of *Geotrichum candidum*) regardless of the library utilized, which is markedly below the manufacturer recommended threshold of 2.00 for a valid identification. The number of correct identifications among the 706 remaining spectra (i.e., corresponding to the species included in the libraries) and the corresponding LS values were statistically different depending on the mass spectra library used for identification (Figures 2 and 3). Notably, the number of identifications concordant with the molecular biology or microscopic identification and LS values significantly increased when the library included an increased number of both RMS per strain and strains per species. In contrast, constructing RMS from 40 raw spectra (B5) instead of 10 raw spectra or reducing the number of raw spectra used to build RMS of the B1 library from 10 to 4 (B0) failed to significantly alter the performance of the identification process (Table 3, Figure 3). Overall, the best results were obtained using library B7, which involved the combination of the highest number of RMS per strain and the highest number of strains per species. Using this library, we obtained 611 (87%) concordant identifications, with LS values higher than 1.700 in 80.85% (494/611) of the cases and LS values higher than 2.000 in 50.90% (311/611) of the cases. Conversely, all 91 (13%) non-concordant identifications exhibited LS values less than 1.700, a value under which the results of LS identification should not be taken in account. These results were dramatically improved compared with those obtained using library B1, which included only one isolate per species and one subculture per isolate. Indeed, using the B1 library, we only obtained 449 (64%) concordant identifications, 40.09% of which displayed LS values higher than 1.7 (180/449) and only 15.59% were higher than 2.000 (70/449). Modulation of the MSP creation parameters, while considering the B1 library, tended to show that the performance of the database could be improved by an increased peak frequency minimum, regarding the number of concordant identifications and the Log Score of the first identification (LS1) mean value. However, when these parameters were applied to the B7 library, we observed the opposite result (Table 4).

**Table 3:** The performance of the libraries considering the species and treatment applied to the clinical quadruplicates

		**Independent spots**								**Best of 4 spots**								**Clinical isolate MSP for 4 spots**							
**Species**		**B0**	**B1**	**B2**	**B3**	**B4**	**B5**	**B6**	**B7**	**B0**	**B1**	**B2**	**B3**	**B4**	**B5**	**B6**	**B7**	**B0**	**B1**	**B2**	**B3**	**B4**	**B5**	**B6**	**B7**
*Alternaria alternata* (10)	a	70	73	80	88	88	83	95	95	60	70	90	90	80	90	100	100	80	80	80	80	80	70	100	100
	b	1.67	1.83	1.84	1.82	1.78	1.71	1.91	1.95	1.91	1.96	1.87	1.89	1.81	1.79	1.98	2.02	1.63	1.7	1.81	1.84	1.74	1.77	1.85	1.92
*Aspergillus flavus* (8)	a	91	78	81	88	88	94	94	100	88	88	100	100	100	100	100	100	88	75	63	63	75	88	88	100
	b	1.58	1.64	1.68	1.73	1.65	1.72	1.74	2.01	1.8	1.76	1.73	1.77	1.73	1.8	1.83	2.09	1.54	1.66	1.93	1.95	1.77	1.77	2.02	2.03
*Aspergillus fumigatus* (85)	a	84	79	84	88	86	85	96	97	92	91	91	91	93	89	98	98	88	85	87	87	86	88	99	98
	b	1.58	1.59	1.67	1.77	1.7	1.71	2.03	2.04	1.69	1.69	1.77	1.87	1.78	1.82	2.13	2.14	1.58	1.6	1.67	1.76	1.69	1.64	2.05	2.08
*Aspergillus nidulans* (2)	a	29	14	14	43	57	29	14	43	50	50	50	50	100	50	50	50	50	50	50	50	100	50	50	50
	b	1.37	1.89	1.89	1.56	1.53	1.39	1.89	1.82	1.58	1.89	1.89	1.89	1.52	1.49	1.89	1.89	1.64	1.62	1.62	1.63	1.41	1.14	1.63	1.83
*Aspergillus niger* (12)	a	85	83	81	77	65	63	77	83	92	83	83	83	67	67	83	83	83	83	75	75	75	75	92	83
	b	1.56	1.57	1.59	1.66	1.54	1.55	1.77	1.89	1.67	1.67	1.68	1.73	1.69	1.71	1.83	1.97	1.53	1.47	1.58	1.65	1.57	1.47	1.6	1.89
*Aspergillus terreus* (10)	a	28	25	33	35	28	25	55	63	30	30	40	40	40	40	60	70	50	40	50	50	50	40	70	70
	b	1.23	1.14	1.19	1.3	1.22	1.22	1.67	1.61	1.35	1.29	1.36	1.41	1.41	1.35	1.79	1.7	1.06	1.17	1.14	1.2	1.21	1.24	1.66	1.66
*Beauveria bassiana* (1)	a	0	0	100	100	75	75	75	75	0	0	100	100	100	100	100	100	0	0	0	0	0	0	0	0
	b			1.2	1.2	1.05	0.93	1.24	1.26			1.32	1.32	1.12	1.06	1.32	1.32								
*Fusarium oxysporum* (2)	a	100	100	100	100	100	100	100	100	100	100	100	100	100	100	100	100	100	100	100	100	100	100	100	100
	b	1.93	2.06	2.06	2.07	1.82	1.78	2.11	2.12	2	2.11	2.11	2.11	1.98	2	2.16	2.17	1.97	2.06	2.06	2.06	1.79	1.9	2.06	2.06
*Microsporum audouinii* (10)	a	45	33	30	30	40	33	30	65	60	50	50	50	40	40	50	70	50	50	50	50	30	40	50	80
	b	1.49	1.4	1.44	1.57	1.35	1.47	1.59	1.8	1.59	1.54	1.55	1.7	1.64	1.67	1.7	1.91	1.41	1.2	1.38	1.45	1.59	1.33	1.54	1.71
*Microsporum canis* (1)	a	0	0	0	0	0	0	25	50	0	0	0	0	0	0	0	100	0	0	0	0	0	0	0	100
	b							1.17	1.51								1.56								1.65
*Penicillium aurantiogriseum*/*chrysogenum* (8)	a	34	34	44	63	41	28	75	75	38	25	50	63	50	38	75	75	50	0	0	0	0	0	38	50
	b	1.7	1.59	1.58	1.88	1.64	1.88	1.98	2	1.86	2.1	1.72	2.03	1.65	1.87	2.19	2.19	1.75						2.07	2.11
*Paecilomyces variotii* (1)	a	0	0	0	0	0	0	25	25	0	0	0	0	0	0	100	100	0	0	0	0	0	0	0	100
	b							1.2	1.28							1.2	1.28								1.76
*Rhizopus oryzae* (3)	a	58	50	58	75	50	58	75	75	67	67	100	100	33	67	100	100	67	67	100	100	67	67	67	67
	b	1.64	2.14	2.05	2.05	1.89	1.69	2.05	2.05	2.03	2.15	1.95	2.06	2.28	1.92	2.06	2.06	1.89	2.16	1.84	1.92	2.02	1.75	2.27	2.27
*Scedosporium apiospermum* (8)	a	47	44	41	47	44	41	56	66	50	50	50	63	38	38	50	63	50	50	63	63	38	63	63	75
	b	1.53	1.33	1.45	1.56	1.59	1.52	1.62	1.67	1.69	1.63	1.65	1.71	1.96	1.81	1.84	1.9	1.97	1.83	1.88	1.9	2.26	1.81	1.96	2.02
*Trichophyton mentagrophytes* (2)	a	88	63	100	100	88	100	100	100	100	50	100	100	50	100	100	100	100	50	100	100	50	100	100	100
	b	1.91	1.93	1.9	1.93	1.8	1.67	1.94	1.94	1.92	2.37	1.97	2.01	2.44	1.74	2.01	2.01	1.84	2.35	1.96	2.01	2.47	1.69	2.01	2.01
*Trichophyton rubrum* (8)	a	47	50	53	69	53	53	78	69	50	75	88	75	63	63	88	75	63	50	63	63	63	38	63	50
	b	1.11	1.13	1.28	1.43	1.47	1.29	1.51	1.65	1.52	1.27	1.35	1.5	1.64	1.54	1.63	1.83	1.35	1.07	1.38	1.46	1.47	1.81	1.54	1.74
*Trichophyton soudanense* (6)	a	17	17	71	38	46	13	92	88	0	17	67	50	50	0	100	100	0	50	67	50	50	17	83	100
	b	1.08	1.24	1.39	1.46	1.37	1.17	1.91	1.97		1.35	1.48	1.53	1.42		2	2.02		0.96	1.03	1.08	1.21	0.86	2	1.9
All species in the library (177)	a	68	64	70	74	70	67	83	87	73	72	80	80	75	72	88	90	73	69	72	72	69	68	84	88
	b	1.56	1.58	1.64	1.73	1.65	1.65	1.92	1.96	1.7	1.7	1.73	1.82	1.75	1.78	2.01	2.05	1.58	1.57	1.64	1.72	1.67	1.63	1.94	2
Non-*A*. *fumigatus* species included in the library (92)	a	53	50	57	61	56	51	71	77	55	55	70	70	59	57	78	83	60	54	59	58	53	51	71	78
	b	1.54	1.57	1.59	1.68	1.58	1.58	1.78	1.86	1.72	1.7	1.67	1.76	1.7	1.71	1.87	1.95	1.57	1.52	1.6	1.66	1.64	1.61	1.81	1.9

a: percentage of concordant identification, b: LS mean of the concordant identifications.

**Table 4:** Modulation of the database performance for independent spots regarding the MSP creation parameters

**Libraries**	**LS1 mean**	**Nb**. **of concordant identifications**	**LS1 mean of concordant identifications**	**Nb**. **of non**-**concordant identifications**	**LS1 mean of non** -**concordant identifications**	**Mean of the difference between LS1 and LS2**	**Max frequency parameter (%)**	**Nb**. **of peaks parameter**
**B1**	1.34	449	1.58	361	1.04	0.38	25	70
**B1b**	1.34	449	1.58	361	1.04	0.38	25	100
**B1c**	1.34	449	1.58	361	1.04	0.38	50	70
**B1d**	1.36	473	1.60	337	1.03	0.40	50	100
**B1e**	1.34	449	1.58	361	1.04	0.38	75	70
**B1f**	1.32	445	1.56	365	1.03	0.36	75	100
**B1g**	1.39	473	1.63	337	1.05	0.43	100	70
**B1h**	1.39	473	1.63	337	1.05	0.43	100	100
**B7**	1.80	611	1.96	199	1.30	0.53	25	70
**B7g**	1.80	595	1.96	215	1.35	0.50	100	70

Considering *Aspergillus fumigatus* isolates separately, the results ranged from 79% (B0/B1) to 97% (B7) concordant identifications, whereas for other species, the percentage of concordant identification ranged from 56% (B0/B1) to 79% (B7) (Table 3). Finally, the identification of a clinical isolate, regardless of the species, was not improved by creating metaspectra (MSP) of the 4 spectra for the comparison of the various libraries (Table 3).

The multivariate analysis findings (Table 5) indicate that concordant identification rates increased significantly with the number of both RMS per strain and raw spectra per RMS. Similarly, the LS values significantly increased (p<10^-4^) with the independent effect of the numbers of RMS per strain and raw spectra per RMS (data not shown). The independent effect of the number of raw spectra per RMS was weaker than the effect of the number of RMS per strain. The percentage of concordant identifications significantly increased exclusively when the number or raw spectra per RMS exceeded 20 (i.e., 40 raw spectra per RMS).

**Table 5:** Multivariate analysis of the factors influencing the probability of the concordant identification of clinical isolates

		**OR**	**95% Confidence Interval**		**P**
**Nb**. **raw spectra**/**RMS**					
	**40 vs**. **10**	1.1767	1.0503 to 1.3183		0.0050
	**40 vs**. **20**	1.2007	1.0705 to 1.3466		0.0018
	**20 vs**. **10**	0.9800	0.8933 to 1.0752		0.6698
**Nb**. **RMS**/**strain**					
	**4 vs**. **1**	1.3362	1.1929 to 1.4968	<10^-4^	
	**4 vs**. **2**	1.1016	1.0122 to 1.1988		0.0250
	**2 vs**. **1**	1.2130	1.0950 to 1.3437		0.0002
**Nb**. **strains**/**species**					
	**3 vs**. **1**	1.2229	1.1173 to 1.3385	<10^-4^	
	**3 vs**. **2**	1.0602	1.0095 to 1.1135		0.0193
	**2 vs**. **1**	1.1534	1.0683 to 1.2453		0.0003

RMS: reference mass spectrum in the library constructed from several raw spectra. Nb.: number.

## Discussion

In contrast with recurrent efforts to improve the reproducibility of the MS-based identification of filamentous fungi by standardizing the pre-treatment procedures, we report the first study aiming to improve identification by comparing the effectiveness of distinct RMS library architectures. However, in a recently published study aiming to identify filamentous fungi using MS, de Carolis et al. [22] have shown that some of the mass spectra data obtained during routine diagnosis matched preferentially with the RMS obtained from either young or mature cultures of the same species. Regarding *Scedosporium* identification, Coulibaly et al. [16] have shown that both the culture media and the duration of culture had a significant impact on MALDI-TOF assay results. However, the standard recommendation to address problems associated with the heterogeneity of microorganism species is merely to increase the number of strains per species in the library. Our findings confirm this hypothesis; however, it is particularly challenging to increase the number of well-characterized strains included in the RMS library for each fungal species. Numerous species have been described to play a role in human infections and, in many cases, only a single strain or a few strains of the same species are preserved in international collections. In the current study, we demonstrated that increasing the number of mass spectra generated from distinct subcultures of a given strain yields a significant improvement in the process of filamentous fungi identification and can partially offset the relatively low number of specific strains available to construct RMS libraries. Modulating MSP creation parameters yielded discrepant results depending on the database that was taken into account. As the B7 database appears ideal for filamentous fungi identification, Bruker’s default parameters for the MSP creation method seem to be more suitable for library construction.

Conversely, the number of spectra derived from a strain (4, 10, 20, or 40) that were used to construct RMS did not result in a marked improvement of the identification performance. This straightforward optimization of RMS library architecture significantly enhanced the identification effectiveness.

In this study, we used quadruplicates of the clinical samples to test the various RMS libraries. By taking only the spectrum with the highest LS value into account, we observed an increased percentage of concordant identifications (e.g., ranging from 87% to 90% with library B7). In parallel, using the four clinical replicates to construct an MSP and then compare it to the various libraries did not alter the results but instead tended to complicate the procedure, as this cannot be performed with RTC software during routine analyses.

The use of standardized conditions (incubation time, temperature, and culture medium) [10,15-18] reduces filamentous fungi pleomorphism but does not preclude the heterogeneity of the mass spectra derived from a given isolate. For example, Chen et al. [17] have improved the accuracy of *Penicillium* identification by assessing the presence or absence of different species-specific peaks in the mass spectrum data obtained when analyzing *Penicillium* spores; however, separating spores from hyphae significantly complicates the pre-processing step. Conversely, some authors have shown that mass spectra heterogeneity is reduced using non-sporulating hyphae obtained in broth culture conditions [21-23]. Unfortunately, the more stringent the method, the less suited it is for high-throughput routine diagnoses. Furthermore, certain impediments are difficult to avoid in routine culture conditions, such as inter-technician variations, variation in protocol, and minor variations (temperature, humidity, or light), when aiming to standardize such protocols.

## Conclusion

Overall, this study provides useful insight into architecture design of reference MS libraries utilized for the MALDI-TOF MS–based identification of filamentous fungi in routine clinical laboratories. Our results show that both incorporating an increased number of subcultures from each strain and increasing the number of strains representing each species are key to improve the architecture of RMS libraries. These findings should be taken into account to construct a more effective library in clinical laboratories.

## Methods

### Fungal strains

The 90 reference filamentous fungus strains corresponding to 30 distinct species that were used to construct the eight distinct reference mass spectrum libraries are detailed in Table 6. Of the 90 reference strains, 63 strains were graciously provided by the BCCM/IHEM (Belgian coordinated collection of microorganisms, Scientific Institute of Public Health, Mycology and Aerobiology Section, Brussels, Belgium), and 3 strains were provided by the Pasteur Institute (Paris, France). The remaining 24 strains were clinical isolates from the Marseille University Hospital mycology laboratory, which were accurately identified via DNA sequence analysis as described below. All strains used to construct the reference database are preserved in the BCCM/IHEM collection. The identification performance of each reference library was tested using 200 clinical isolates from the Marseille University Hospital mycology laboratory.

**Table 6:** Details of the 90 reference strains corresponding to 30 filamentous fungi species of medical interest included in the reference libraries

**Species**	**Strain**	**Source**	**Included in library(ies)**
*Absidia corymbifera*	IHEM16288	BCCM/IHEM	B0-B7
	IHEM14734	BCCM/IHEM	B6-B7
	IHEM21658	BCCM/IHEM	B7
*Acremonium strictum*	Pasteur 173	Pasteur Institute	B7
	IHEM19179	BCCM/IHEM	B0-B7
	IHEM 22371	BCCM/IHEM	B6-B7
*Acrophialophora fusispora*	IHEM15939	BCCM/IHEM	B0-B7
	IHEM19591	BCCM/IHEM	B6-B7
	IHEM19730	BCCM/IHEM	B7
*Alternaria alternata*	IHEM22669	BCCM/IHEM	B6-B7
	IHEM21999	BCCM/IHEM	B0-B7
	IHEM9788	BCCM/IHEM	B7
*Aspergillus candidus*	IHEM15975	BCCM/IHEM	B6-B7
	IHEM14607	BCCM/IHEM	B7
	IHEM9678	BCCM/IHEM	B0-B7
*Aspergillus flavus*	IHEM23376	BCCM/IHEM	B6-B7
	IHEM14475	BCCM/IHEM	B0-B7
	AFLA002	clinical strain	B7
*Aspergillus fumigatus*	IHEM15161	BCCM/IHEM	B0-B7
	IHEM19416	BCCM/IHEM	B6-B7
	IHEM22145	BCCM/IHEM	B7
*Aspergillus nidulans*	IHEM23179	BCCM/IHEM	B0-B7
	IHEM23366	BCCM/IHEM	B6-B7
	Pasteur 123	Pasteur Institute	B7
*Aspergillus nigri section*	IHEM9673	BCCM/IHEM	B0-B7
	IHEM5077	BCCM/IHEM	B6-B7
	ANIG001	clinical strain	B7
*Aspergillus terreus*	IHEM17777	BCCM/IHEM	B0-B7
	IHEM18939	BCCM/IHEM	B6-B7
	IHEM9995	BCCM/IHEM	B7
*Beauveria bassiana*	IHEM6954	BCCM/IHEM	B6-B7
	Pasteur 146	Pasteur Institute	B7
	IHEM18747	BCCM/IHEM	B0-B7
*Exophiala dermatitidis*	EXOP001	clinical strain	B7
	IHEM23421	BCCM/IHEM	B0-B7
	IHEM9780	BCCM/IHEM	B6-B7
*Fusarium oxysporum*	IHEM18448	BCCM/IHEM	B0-B7
	FUSA003	clinical strain	B7
	IHEM20619	BCCM/IHEM	B6-B7
*Fusarium solani*	IHEM22015	BCCM/IHEM	B0-B7
	IHEM7504	BCCM/IHEM	B6-B7
	1000694	clinical strain	B7
*Fusarium verticillioides*	IHEM20180	BCCM/IHEM	B6-B7
	IHEM22962	BCCM/IHEM	B7
	IHEM18495	BCCM/IHEM	B0-B7
*Microsporum audouinii*	013	clinical strain	B0-B7
	027	clinical strain	B6-B7
	169	clinical strain	B7
*Microsporum canis*	05-05-00921	clinical strain	B0-B7
	249	clinical strain	B6-B7
	1100085	clinical strain	B7
*Microsporum gypseum*	05-05-00961	clinical strain	B0-B7
	MO	clinical strain	B6-B7
	93629	clinical strain	B7
*Paecilomyces variotii*	IHEM17703	BCCM/IHEM	B6-B7
	IHEM3285	BCCM/IHEM	B7
	IHEM16627	BCCM/IHEM	B0-B7
*Penicillium aurantiogriseum*	IHEM18723	BCCM/IHEM	B0-B7
	IHEM20176	BCCM/IHEM	B6-B7
	IHEM20357	BCCM/IHEM	B7
*Penicillium chrysogenum*	IHEM17894	BCCM/IHEM	B0-B7
	IHEM22667	BCCM/IHEM	B6-B7
	IHEM20859	BCCM/IHEM	B7
*Rhizomucor pusillus*	IHEM21236	BCCM/IHEM	B0-B7
	IHEM18686	BCCM/IHEM	B6-B7
	IHEM16462	BCCM/IHEM	B7
*Rhizopus oryzae*	IHEM13186	BCCM/IHEM	B0-B7
	IHEM21660	BCCM/IHEM	B7
	IHEM16287	BCCM/IHEM	B6-B7
*Scedosporium apiospermum*	IHEM3817	BCCM/IHEM	B7
	IHEM6908	BCCM/IHEM	B6-B7
	IHEM14632	BCCM/IHEM	B0-B7
*Scedosporium prolificans*	IHEM5739	BCCM/IHEM	B0-B7
	IHEM18755	BCCM/IHEM	B6-B7
	IHEM22339	BCCM/IHEM	B7
*Scopulariopsis brevicaulis*	IHEM15574	BCCM/IHEM	B0-B7
	IHEM1690	BCCM/IHEM	B6-B7
	IHEM22982	BCCM/IHEM	B7
*Trichophyton mentagrophytes*	1036279	clinical strain	B0-B7
	1039316	clinical strain	B6-B7
	5-97-00858	clinical strain	B7
*Trichophyton rubrum*	60D	clinical strain	B0-B7
	184	clinical strain	B6-B7
	235	clinical strain	B7
*Trichophyton roseum*	IHEM1535	BCCM/IHEM	B0-B7
	7941	clinical strain	B6-B7
	IHEM2478	BCCM/IHEM	B7
*Trichophyton soudanense*	243	clinical strain	B0-B7
	095	clinical strain	B6-B7
	1100082	clinical strain	B7

BCCM/IHEM: Scientific Institute of Public Health, Mycology & Aerobiology Section, Brussels, Belgium; the characteristics of the mass spectra libraries B0 to B7 are described in Table 7.

**Table 7:** Architectural characteristics of the reference libraries

**Library number**	**Number of raw spectra per RMS**	**Number of RMS per strain**	**Number of strains per species**	**Library characteristics**
B0	4	1	1	1 RMS4 x 30 strains
B1	10	1	1	1 RMS10 x 30 strains
B2	10	2	1	2 RMS10 x 30 strains
B3	10	4	1	4 RMS10 x 30 strains
B4	20	2	1	2 RMS20 x 30 strains
B5	40	1	1	1 RMS40 x 30 strains
B6	10	4	2	4 RMS10 x 60 strains
B7	10	4	3	4 RMS10 x 90 strains

### Culture

Each reference strain was subcultured on four Sabouraud Gentamicin Chloramphenicol agar plates (AES, France) at 30°C. The strains used to construct the reference libraries and the isolates obtained from clinical samples were analyzed as soon as a fungal colony grew on the agar (usually after 48–72 hours). The clinical isolates were identified via morphological assessment, DNA sequencing, and MALDI-TOF MS as described below.

### Clinical isolate identification

All 200 clinical isolates were identified in parallel by two trained mycologists following the identification keys of the Atlas of Clinical Fungi [24]. If the morphological identification was impossible or conflicted with the MALDI-TOF MS-based identification results, the isolate was further analyzed using DNA sequencing. DNA sequence-based identification was performed by analyzing the ITS 2 (primer ITS3: GCA TCG ATG AAG AAC GCA GC and primer ITS4c: TCC TCC GCT TAT TGA TAT GC) and D1-D2 (primer D1: AAC TTA AGC ATA TCA ATA AGC GGA GGA and primer D2: GGT CCG TGT TTC AAG ACG G) variable regions of the 28S unit of the rRNA gene as described by de Hoog et al. [24]. DNA extraction was performed using a QIAmp DNA kit (QIAGEN, Courtaboeuf, France). The reaction mixture was subjected to 35 cycles of 30 s denaturation at 94°C, 30 s primer annealing at 53°C, and 1 min primer extension at 72°C for the ITS 2 region and 40 cycles of 20 s denaturation at 94°C, 30 s primer annealing at 58°C, and 1 min primer extension at 72°C for the D1-D2 region. The sequencing reactions were performed using the same primers used for amplification. In both cases, the sequencing mixture was subjected to 25 cycles of 10 s denaturation at 96°C, 5 s primer annealing at 50°C, and 4 min primer extension at 60°C. Purification of the sequences was performed using BigDye® XTerminator™ (Applied Biosystems, Inc., Courtaboeuf, France), and the different reactions were processed using a 3130 Genetic Analyzer (Applied Biosystems, Inc., Courtaboeuf, France). The resulting sequences were then compared using the Medical Fungi pairwise sequence alignment tool (http://www.cbs.knaw.nl/Medical/BioloMICSSequences.aspx). Identification was validated when the sequence was at least 300 nucleotides long and the similarity percentage was over 98%.

### Raw mass spectra acquisition

The colonies were gently scraped with sterile plastic pliers to obtain an aliquot (approximately 3–4 mm in diameter) of fungal spores and hyphae. This sample was first suspended in 75% ethanol HPLC. Next, the hydro-alcoholic solution was removed via 10 min centrifugation at 13,000 g, and the pellet was suspended in 10 μL of 70% formic acid (Sigma-Aldrich, France) by vigorously pipetting the sample up and down. After a 5-min incubation, 10 μL of acetonitrile HPLC (VWR International S.A.S., Fontenay-sous-Bois, France) was added, and the mixture was incubated at room temperature for 5 min. Finally, the sample was centrifuged for 2 min at 13,000 g. One microliter of the supernatant (consisting of a mixture of fungal proteins) was deposited for each reference strain subculture in 10 replicates on a polished steel target (MTP384, Bruker Daltonics GmbH, Bremen, Germany) and air-dried. Each deposit was then covered with 1 μL of a freshly prepared solution of α-cyano-4-hydroxycinnamic acid (HCCA) in 50% acetonitrile HPLC (VWR International S.A.S., Fontenay-sous-Bois, France) and 2.5% trifluoroacetic acid HPLC (TFA) matrix (Applied Biosystems®, Villebon sur yvette, France) [21]. The spectra were acquired after 650 shots in linear mode using an UltrafleXtreme™ instrument (Bruker Daltonics, Germany) in the ion-positive mode with a 337-nm nitrogen laser. The following adjustments were used: delay, 170 ns; ion source 1 voltage, 20 kV; ion source 2 voltage, 18.5 kV; mass range, 3–20 kDa; and measuring raster: spiral_small. An *E*. *coli* calibration was performed before every experiment using a Bruker Bacterial Test Standard (Bruker Daltonics GmbH, Bremen, Germany). The data were automatically acquired using the AutoXecute function of the FlexControl v2.4 software and then exported into MALDI Biotyper v2.1 (Bruker Daltonics) software. Only the peaks with a signal/noise ratio ≥10 were considered.

### Constructing the reference mass spectra (RMS)

The RMS were established by combining i) 4 raw spectra obtained from one subculture (RMS4); ii) 10 raw spectra obtained from one subculture (RMS10); iii) 20 raw spectra, 10 from two subcultures each (RMS20); or iv) 40 raw spectra, 10 from four subcultures each (RMS40) of a given reference strain using the “MSP creation” function of the MALDI Biotyper v2.1 software (Table 7). The following settings were applied (Bruker’s default parameters): Max. Mass Error of each single spectrum: 2000; Desired Mass Error of the MSP: 200; Desired Peak Frequency Minimum: 25%; and Max. Desired Peak Number of the MSP: 70. The modulation of the number of peaks and desired peak frequency minimum of the MSP creation parameters has been tested regarding the B1 library, and the modified parameters were tested on the B7 database (Table 4).

### Architecture of the eight mass spectral libraries

The same fungal species were included in the eight libraries that differed in number of raw spectra used to build the RMS (described above), RMS included for each reference strain, and strains included. The characteristics of the various libraries are detailed in Table 2.

### MALDI-TOF MS–based identification of clinical isolates

Raw mass spectra were obtained from clinical isolates using the same procedure as for the reference strains with the exception that the supernatant were deposited in quadruplicate. The deposits, referred to as spots 1, 2, 3, and 4, correspond to the first, second, third, and fourth extraction supernatant deposit of each sample, respectively. The raw MS data for each spot was successively matched to the eight reference libraries, and the resulting “best match” LS values were calculated using MALDI Biotyper software. An alternate identification process was assessed by constructing an MSP with the four spots corresponding to each of the clinical isolates and comparing isolate MSP with each of the RMS in the libraries.

The interpretation of the results was initially performed independently of the LS value. If the MS identification was identical to the microscopic identification or the sequencing analysis results, the identification was considered concordant, regardless of the LS value; otherwise, it was considered a non-concordant identification. Next, the LS value was considered to be applicable in comparing the performance of the various libraries. As approximately half of the clinical isolates corresponded to the *Aspergillus fumigatus* species, a comparison was also performed between the libraries when either considering or disregarding this dominant species.

Library performance was also compared regarding the method by which the clinical quadruplicates were considered as follows: i) each spectrum was treated independently, ii) only the spectrum with the highest LS was taken into account, regardless of whether it was concordant, and iii) an MSP of the four spectra was constructed, and the clinical MSP was compared to each library.

### Ambiguous MS identifications

Some of the species included in this study are known to be difficult to distinguish, even via ITS sequencing. Reference spectra were included in the libraries, but concordance could neither be confirmed nor contradicted. The species included were *Penicillium aurantiogriseum* and *Penicillium chrysogenum*. Both MS identifications were then considered concordant with the other identification methods.

### Reference mass spectra library architecture assessment

Analyzing 200 clinical isolates, we tested the influence of the number of the following parameters on identification effectiveness: i) raw spectra used to build a reference MS, ii) reference MS included per strain, and iii) strains per species included in the library. The various reference spectrum architectures were compared with respect to the number of correct and false identifications as well as the mean LS values of both correct and false identifications.

### Statistical analysis

The concordant and non-concordant identification results were compared two by two using the paired and non-parametric McNemar’s test. The results of the quantitative variable LS analysis were compared using the non-parametric rank sum test of the Kruskall-Wallis test. When the results of the Kruskall-Wallis test indicated a statistical difference between the LS values derived from the different mass spectral libraries, a post hoc statistical analysis was performed, which involved a pairwise comparison of the LS values obtained from each library using the Wilcoxon signed-rank test with Bonferroni adjustment. These analyses were performed using R software (http://www.r-project.org/) with the MASS and ROCR packages. To further examine the influence of library architecture on the probability of obtaining a correct identification, a multivariate analysis was conducted with the Genmod procedure of the SAS 9.2 (Cary, NC, USA) statistical software using the generalized estimating equations option to account for the non-independence of identification results obtained from the same isolate tested against distinct libraries. These analyses were performed to identify the optimal reference library architecture; therefore, the results obtained with isolates for which the species was not included in the library were excluded from this multivariate analysis. All statistical tests were two-sided with a p≤ 0.05 significance level.

### Availability of supporting data

These data are included in Table 6 entitled “Details of the 90 reference strains included in the reference libraries”.

## Abbreviations

MALDI-TOF: Matrix-assisted laser desorption/ionization time-of-flight; MS: Mass Spectrometry; RMS: Reference Meta Spectrum; MSP: Meta spectrum; LS: Log Score; PCR: Polymerase Chain Reaction; DNA: Deoxyribonucleic acid; rRNA: Ribosomal ribonucleic acid; s: Second; °C: Temperature in Celsius; min: Minute; μL: Microliter; nm: Nanometer; ns: Nanosecond; kV: Kilovolt; kDa: Kilodalton; MSL: Mass spectra library; Nb.: Number.

## Competing interests

The authors declare that they have no competing interests.

## Authors’ contributions

ACN, CC, CL, PF, SR, and MH performed the experiments. ACN, CC, SR, and RP conceived the study, analyzed the data, and wrote the manuscript. CC and SR carried out the statistical analyses. ACN and CC prepared the figures and tables. All authors read and approved the final manuscript.
